# *Hericium erinaceus*, in combination with natural flavonoid/alkaloid and B_3_/B_8_ vitamins, can improve inflammatory burden in Inflammatory bowel diseases tissue: an *ex vivo* study

**DOI:** 10.3389/fimmu.2023.1215329

**Published:** 2023-07-03

**Authors:** Antonietta Gerarda Gravina, Raffaele Pellegrino, Giovanna Palladino, Annachiara Coppola, Giovanni Brandimarte, Concetta Tuccillo, Fortunato Ciardiello, Marco Romano, Alessandro Federico

**Affiliations:** ^1^ Hepatogastroenterology Unit, Department of Precision Medicine, University of Campania “Luigi Vanvitelli”, Naples, Italy; ^2^ Division of Internal Medicine and Gastroenterology, Cristo Re Hospital, Rome, Italy; ^3^ Medical Oncology Unit, Department of Precision Medicine, University of Campania “Luigi Vanvitelli”, Naples, Italy

**Keywords:** inflammatory bowel disease, *Hericium erinaceus*, quercetin, berberin, biotin, niacin, Crohn’s disease, ulcerative colitis

## Abstract

*Hericium erinaceus*, berberine, and quercetin are effective in experimental colitis. It is unknown whether they can ameliorate inflammatory bowel diseases in humans. This *ex vivo* study aimed to evaluate the anti-inflammatory potential of a nutraceutical compound of HBQ-Complex^®^ (*H. erinaceus*, berberine, and quercetin), biotin, and niacin in inflammatory bowel disease patients. Tissue specimens were obtained either from Normal-Appearing Mucosa (NAM) or from Inflamed Mucosa (IM) in 20 patients with inflammatory bowel disease. mRNA and protein expression of COX-2, IL-10, and TNF-α were determined in NAM and IM biopsy samples (T0). IM samples were then incubated in HBQ-Complex^®^ (with the addition of niacin and biotin), and COX-2, IL-10, and TNF-α tissue levels were evaluated at 120 minutes (T1) and 180 minutes (T2). Incubation with this compound resulted in a progressive decrease in gene and protein COX-2 and TNF-α expression at T1/T2 in the IM. IL-10 showed an opposite trend, with a progressive increase of mRNA and protein expression over the same time window. HBQ-Complex^®^ (with the addition of niacin and biotin) decreased the expression of proinflammatory cytokines at the mRNA and protein levels in IBD tissue. On the contrary, mRNA and protein expression of the anti-inflammatory cytokine IL-10 showed a progressive increase.

## 1 Introduction

Inflammatory Bowel Diseases (IBD) are characterized by sustained chronic gastrointestinal inflammation and include mainly Crohn’s Disease (CD) and Ulcerative Colitis (UC) (1).

*Hericium erinaceus* (*H. erinaceus*, class *Agaricomycetes*, phylum *Basidiomycota*) is a Chinese medicinal, edible fungus mainly distributed in East Asian regions with a centuries-old history in traditional Chinese medicine (2). This natural compound has shown efficacy in several gastrointestinal disorders. In detail, it has been used as a home remedy for conditions such as chronic gastritis, peptic ulcers, colitis, and dyspepsia (3). Also, *H. erinaceus* has been demonstrated to exert immunomodulatory and antioxidant effects (2). Moreover, *H. erinaceus* decreases the production of prostaglandin E2 and reactive oxygen species and suppresses the expression of proinflammatory genes by inhibiting the p65 subunit of Nuclear Factor kappa-light-chain-enhancer of activated B cells (i.e., NF-κB) in macrophage cell lines (4). Among proinflammatory cytokines, Tumor Necrosis Factor-alpha (TNF-α) produced by activated intestinal macrophages is one of the main targets of monoclonal antibody-based therapies (5). While experimental evidence suggests that *H. erinaceus* is an effective natural anti-inflammatory agent, studies on its effects in IBD patients are lacking. Diling et al. conducted a pre-clinical study in a colitis (2,4,6-trinitrobenzene sulfonic acid-based) mouse model, showing an increase of interleukin (IL)-10 levels and a reduction of TNF-α levels after fourteen days of treatment with extracts of *H. erinaceus* with suppression of NF-κB p65 (6). A few other pre-clinical experiments in mouse IBD models have shown similar results, focusing mainly on polysaccharide extracts (i.e., EP-1) of *H. erinaceus* (7, 8). In particular, EP-1 has shown the ability to increase the activity of superoxide dismutase enzymes in an *in vivo* experimental colitis model, thereby reducing malondialdehyde and proinflammatory markers (i.e., TNF-α, IL-1, and IL-6) (9).

In contrast, Xie et al. evaluated the administration of *H. erinaceus* as a dietary supplement in a small group of healthy volunteers, observing an increase in the alpha diversity of the gut microbiota by increasing the availability of short-chain fatty acid-producing bacteria with concomitant reduction of several pathobionts (10). In addition, *H. erinaceus* has also been shown to improve anxiety-depressive disorders in robust studies such as clinical trials (11, 12), disorders of high prevalence in patients with IBD, and impact the course of the disease (13, 14).

Quercetin, a flavonoid with antioxidant properties, can potentially reduce TNF-α levels and increase IL-10 levels in *C. rodentium*-induced experimental colitis models (15). Berberine, a plant isoquinolone alkaloid found in *Berberis vulgaris* and *Berberis aquifolium*, can modulate cyclooxygenases (COX) by reducing the production of proinflammatory cytokines (16). Finally, in a randomized phase I trial in UC patients, berberine significantly improved the degree of tissue inflammation assessed by the Geboes score by 40% (17). Several soluble vitamins, such as B vitamins (i.e., biotin or niacin), have demonstrated IBD-targeted anti-inflammatory power (18). In addition, a biotin-deficient diet could induce UC-like colitis in mice, while mice with experimental dextran sulfate sodium-induced colitis show biotin deficiency, and, moreover, IBD patients tend to show more pronounced biotin deficiency than healthy controls (19).

Whether a combination of *H. erinaceus*, quercetin, berberine, niacin, and biotin can exert anti-inflammatory effects in IBD in an *ex vivo* experimental model based on a human tissue sample has never been investigated.

Therefore, this study was designed to evaluate whether *H. erinaceus*, berberine, quercetin, niacin, and biotin in combination exert anti-inflammatory effects in an *ex vivo* experimental model of intestinal tissue samples obtained from patients with CD or UC.

The study hypothesizes that exposure of our *ex vivo* IBD model to HBQ-Complex*^®^* (with the addition of niacin and biotin) may reduce the inflammatory load of intestinal tissue affected by the disease (i.e., CD or UC).

## 2 Materials and methods

### 2.1 Study design and population

Two equally distributed groups of IBD patients (i.e., 10 UC and 10 CD patients) at their first endoscopic and histological diagnosis of active CD or UC referred to our Unit of Hepatogastroenterology of the University of Campania “Luigi Vanvitelli” were selected. Patients with clinical, endoscopic, instrumental, and medical history signs suggestive of infectious, ischemic, actinic, or immunotherapy-induced colitis; patients with clinically significant infections in the past six months or with conditions that have resulted in their hospitalization in the past six months; patients who are minors (i.e., < 18 years old), pregnant, or lactating; patients with a both remote and forthcoming personal history of oncological disease; and patients with other non-functional disorders of interest to the gastrointestinal tract (e.g., celiac disease) were excluded. Patients included had to have no clinical, laboratory, or instrumental evidence of comorbidities other than baseline IBD and be naïve to any IBD treatment.

Finally, an additional exclusion criterion was consumption within six months of the start of the study of drugs potentially associated with mucosal toxicity on the gastrointestinal tract (such as nonsteroidal anti-inflammatory drugs) as well as drugs capable of determining impact on the gut microbiota (for example, antibiotics, prebiotics, symbiotics, and probiotics).

According to the Montreal classification, patients with UC had to have an exclusively E1 (ulcerative proctitis) or E2 (left-sided UC) extent of disease. On the other hand, CD patients had to possess either L2 (colon) or L3 (ileocolonic) extension of disease.

In addition to the colonic tissue sampling already performed by clinical practice, patients were given additional sampling in target colonic regions for the study (read further).

### 2.2 Colonic mucosa sampling

Colonic biopsies were performed during colonoscopy [whose indication was previously given by an IBD gastroenterologist according to current guidelines (20)] as previously written in targeted areas, i.e., those affected by disease and those grossly spared by lesions. The definition of disease-affected mucosa, i.e., Inflamed Mucosa (IM), was based on validated scores for both CD and UC. For CD, the Simple Endoscopic Score for CD (SES-CD) (21) was used, and IM was defined by an SES-CD > 2. On the other hand, the Mayo Endoscopic SubScore (ESS) (22) was used for UC, and IM was defined by an ESS > 0. In contrast, areas of Normal-Appearing Mucosa (NAM) were definitive for an SES-CD = 0 and an ESS = 0 (23). Colonoscopies with biopsies were all performed by the same endoscopist who had a considerable number of endoscopic staging procedures in IBD patients to her credit.

### 2.3 IBD activity evaluation at the study inclusion

At the time of inclusion, disease activity was assessed by Partial Mayo Score (PMS) for patients with UC and Harvey-Bradshaw index (HBI) for patients with CD. Specifically, patients with UC were classified as having mild disease if PMS was 2-4 and moderate if between 5-7. Patients in remission (PMS < 2) or with severe activity (PMS > 7) were not included (23).

For CD, the disease was considered mild if HBI was between 5-7 and moderate if between 8-16. Therefore, patients with disease in remission (HBI < 5) or with severe disease (HBI > 16) were not included (23).

### 2.4 Culture of colonic mucosal specimens

After sampling, NAM colonic mucosal specimens and one IM colonic specimen for each patient were frozen at - 70°C. The other IM samples were then cultured in the Dulbecco’s modified Eagle’s medium and placed in a special closed culture chamber and inserted in a dedicated cell incubator with the addition of the compound under our study (under positive oxygen pressure and a controlled temperature of 37°C). The latter consisted of 525 mg of *H. erinaceus* powder (5% polysaccharides) and 225 mg of *H. erinaceus* as an extract (30% polysaccharides), 75 mg of quercetin titled to 98%, 225 µg of biotin, 27 mg of niacin and, finally, 75 mg of Berberis vulgaris titled to 97% (the combination of only H. erinaceus, berberine, and quercetin is referred to as “HBQ-Complex®”).

Four colonic mucosa samplings were performed for each patient (whether CD or UC) to obtain both the NAM sample and three IM samples that could be examined at different timings. These were, in detail, the baseline (T0) after 120 minutes of incubation as previously described (T1) and, finally, after 180 minutes of incubation (T2).

### 2.5 Technical specifications of *H. erinaceus*-derived components

The *H. erinaceus* component 5% polysaccharide powder is extracted from the sporophorum of the fungus. The macroscopic characteristics are fine brown powder. The polysaccharide percentage of the powder is 5% (detected by ultraviolet-visible spectroscopy).

The *H. erinaceus* component, as a 30% polysaccharide extract, is also extracted from the sporophorum of the fungus. It contains 10% maltodextrin as excipient. The solvent used is 100% water, and the extraction ratio is 10:1. The amount of 30% polysaccharides is evaluated by ultraviolet-visible spectroscopy. The macroscopic appearance is of fine yellow-brown powder. It is almost soluble in water. In both extracts, the amount of polycyclic aromatic hydrocarbon is ≤ 50 parts per billion, while the amount of benzo(a)pyrene is ≤ 10 parts per billion (both amounts evaluated by gas chromatography-mass spectrometry). The apparent density of both compounds is 0.4-0.7 g/mL. The sieve analysis is 100% pass 80 mesh. The loss on drying and total ash are ≤ 10%. The total plate count is ≤ 1000 colony-forming units/g. Heavy metals are ≤ 10 mg/Kg in both compounds.

The 5% powder and the 30% polysaccharide extract have no ethylene oxide or undergo irradiation. They contain an amount of gluten < 20 parts per million. By the datasheet, they have a shelf life of 24 months if stored in a tightly closed container away from moisture, light, and oxygen. The compounds for this study were handled following the datasheet and used within the indicated shelf life.

### 2.6 Protein extraction and Western blot analysis

Frozen human colon mucosa samples (i.e., NAM, IM at T0, IM at T1, and IM at T2) were homogenized in RIPA lysis buffer [0.1% sodium dodecyl sulphate (SDS), 0.5% deoxycholate, 1% Nonidet, 100 mM NaCl, 10 mM Tris-HCl (pH 7.4)] containing a protease inhibitor cocktail (Sigma, St Louis, Missouri, USA), 0.5 mM dithiothreitol, and 0.5% phenylme-thylsulphonyl fluoride. After 30 min at 4°C, tissue lysates were clarified by centrifugation at 14,000 rpm for 10 min at 4°C. The cleared tissue lysates were collected and stored at −80°C, and the protein concentration of each sample was determined by Bradford assay (Coomassie brilliant blue protein assay; Bio-Rad, Melville, NY, USA). The antibodies used in this study were as follows: (1) mouse monoclonal anti-TNF-α (sc-52746 Santa Cruz Biotechnology); (2) mouse monoclonal anti-IL-10 (sc-32815 Santa Cruz Biotechnology); (3) mouse monoclonal anti-COX-2 (sc-19999 Santa Cruz Biotechnology); and (4) mouse mono-clonal antibody to α-Tubulin, (No #CPA9108 Cohesion biosciences). The secondary antibody was anti-mouse IgG conjugated to horseradish peroxidase (HRP) (sc -516102 Santa Cruz Biotechnology) as appropriate. Total protein extracts were subjected to SDS–PAGE (10% and 7% polyacrylamide) under reducing conditions. After electrophoresis, proteins were transferred to a nitrocellulose membrane (pure nitrocellulose membrane, 0.45 m Bio-Rad Laboratories); complete transfer was assessed using pre-stained protein standards (Invitrogen LC5925). To block non-specific binding sites, the membranes were treated for 1 h with a blocking solution, namely 5% milk in TNT (10 mM Tris pH 8, 150 mM NaCl and 0.05% Tween-20), and then were incubated overnight at 4°C with the primary antibody in TNT (0.05% Tween-20) 20% FBS. After washing with TBS, membranes were incubated for 2 h (at room temperature) with the appropriate secondary antibody. According to the manufacturer’s instructions, immunoreactive proteins were detected by development with the SuperSignal West Pico Plus Chemiluminescent Substrate Kit (Thermo Scientific, Rockford, USA).

### 2.7 RNA extraction, reverse transcription-polymerase chain reaction analysis, and real-time PCR

Gene expression of TNF-α, IL-10, and COX-2 was evaluated by quantitative RT-PCR analysis. Total RNA was extracted from colonic tissue using a RNeasy Plus Mini Kit (Qiagen, Hilden, Germany). The purity of total RNA was assessed using NanoDrop_ ND-100 spectrophotometer at 260 nm. Two micrograms of total RNA were used in the first strand cDNA synthesis by TaqMan*^®^* Reverse Transcription Reagents (Applied Biosystems, Branchburg, NJ, USA). The cDNA was diluted with RNase-free water for a final volume di 200 µl and stored at - 20°C until used gene expression levels were analyzed by Taq-Man*^®^* Gene Expression Assays (Applied Biosystems). Quantitative Real-Time PCR was carried out in triplicate using a pre-optimized primer/probe mixture and TaqMan universal PCR master mix (Applied Biosystems) on a StepOne TM Real-Time PCR System (Applied Bio-systems). The GAPDH housekeeping gene was used as an endogenous control for normalizing gene expression assays. The sample values represent X-fold differences from a control sample (given a designated value of 1) within the same experiment. The assay identification (Assay ID) for each gene is as follows: TNF-α (Hs02621508_s1), IL-10 (Hs00961622_m1), COX-2 (Hs00153133_m1), and GAPDH (Hs02786624-g1).

### 2.8 Statistical analysis

Descriptive statistics were used for data presentation. Continuous variables were exhibited as median (interquartile range). Comparison of gene PCR expression levels, obtained by the ^ΔΔ^Ct method, between different study groups (i.e., NAM and IM) was performed with the Mann-Whitney-U-test. In addition, the Wilcoxon-signed ranks test was employed to evaluate the changes in these gene PCR expression levels between different study times (i.e., T0, T1, and T2). Two-tailed p-values lower than 0.05 were considered significant by setting the α error equal to 0.05. The p-values were expressed by also reporting the 95% confidence interval. Statistical analyses were performed using IBM*^®^* SPSS*^®^*, while graphical representations were performed using GraphPad Software, Inc.

## 3 Results

### 3.1 Characteristics of included patients

Ten (50%) patients with CD and 10 (50%) patients with UC were included in the study. Of these, 11 were male (55%) and nine were female (45%). Regarding anthropometric data, the weight of the included patients was 78.5 (74 - 84.5) kg. Height was 170.5 (163.5 - 178) cm, and BMI was 27.8 (25.94 - 29.35) Kg/m2. Weight (p=0.276; 95% CI 0.267 - 0.285), height (p=0.761; 95% CI 0.752 - 0.769), and BMI (p=0.651; 95% CI 0.642 - 0.660) were not different according to the type of IBD (i.e., CD or UC). Regarding disease extension, all patients with CD had L3 extension, while regarding patients with UC, nine (90%) had E2 extension, while one (10%) had E1. The median age was 35 years. None of the patients smoked, consumed alcohol, or had a family history of IBD. In addition, none of the patients had undergone surgery for the underlying IBD or other comorbidities. Only one UC patient reported prior antibiotic use (i.e., cefixime to treat pharyngitis a year before inclusion in the study). None of the patients had additional comorbidities compared with baseline IBD.

Regarding IBD activity, the PMS overall was 5 (2.75 - 6.25), while the HBI was 8 (5.75 - 9.75). In detail, stratifying the data by the degree of disease activity in UC patients, four (40%) had mild and six (60%) moderate disease. In the CD group, four (40%) had mild and six (60%) moderate IBD activity.

### 3.2 HBQ-Complex^®^ (in combination with biotin and niacin) downregulates the expression of TNF-α and COX-2 and upregulates the expression of IL-10 mRNA levels

**Figure 1:**
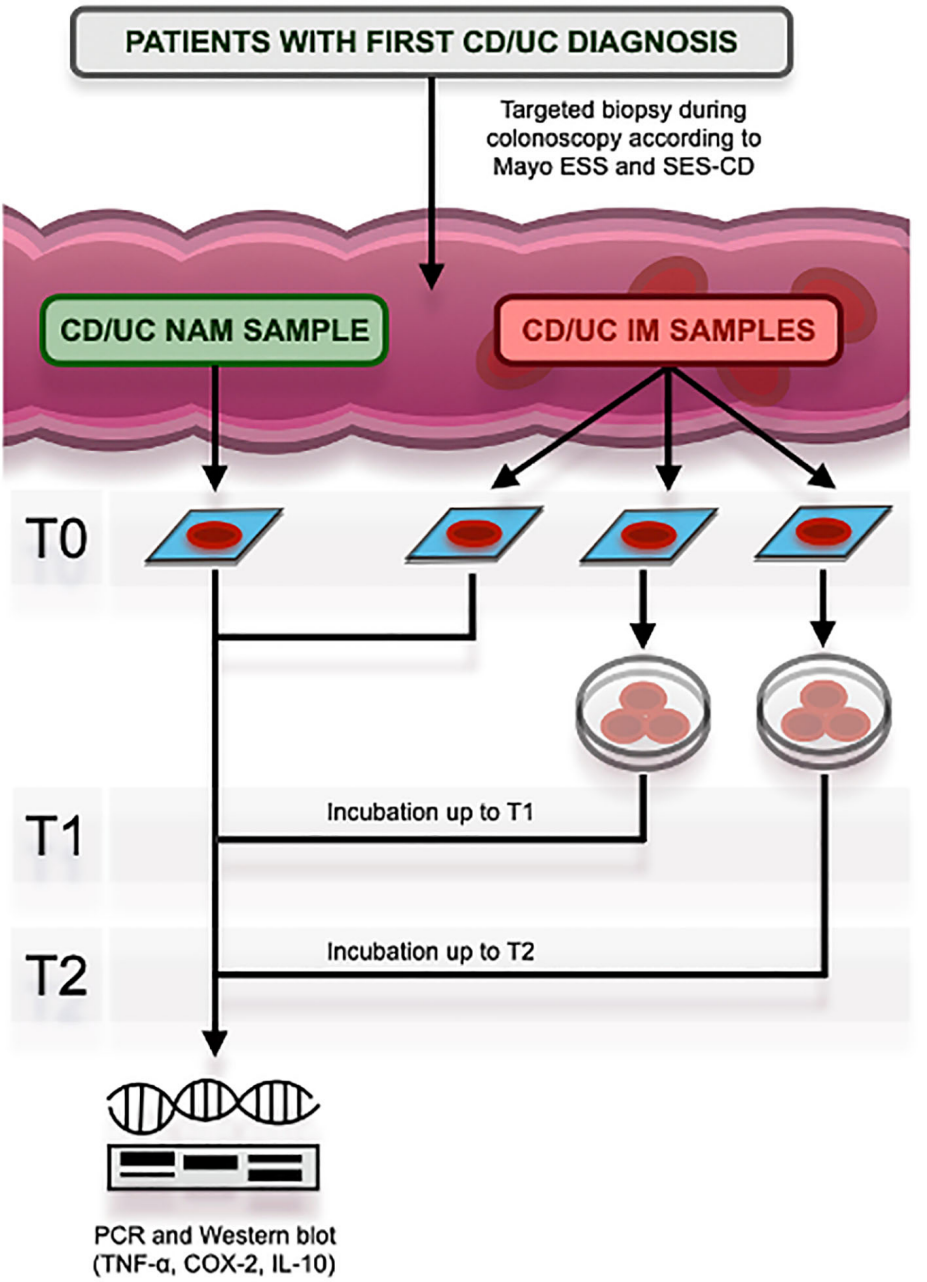
Study protocol at its different times, namely T0 (baseline), T1 (after 120 minutes), and T2 (after 180 minutes). CD, Crohn’s Disease; UC, Ulcerative Colitis; ESS, Endoscopic SubScore; SES-CD, Simple Endoscopic Score for Crohn’s Disease; NAM, Normal Appearing Mucosa; IM, In-flamed Mucosa; PCR, Polymerase Chain Reaction; TNF-α, Tumor Necrosis Factor-alpha; COX-2, Cyclooxygenase type 2; IL-10, Interleukin-10.

To assess the anti-inflammatory impact of HBQ-Complex*^®^* niacin, and biotin, we first evaluated the mRNA levels of three pivotal mediators in the intestinal inflammatory process (i.e., COX-2, IL-10, and TNF-α) in the IM and the NAM obtained from CD and UC patients. The study protocol is summarized in **Figure 1**.

**Figure 2:**
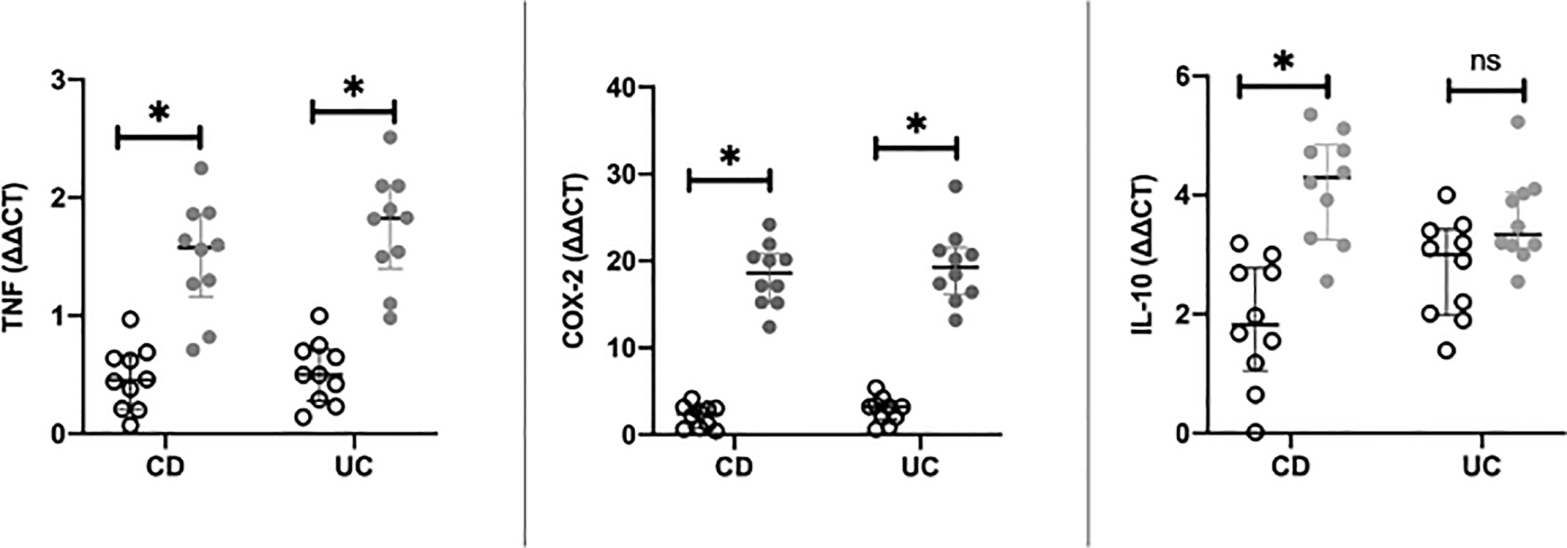
Differences in RT-PCR gene expression expressed as expression ratio for Cyclooxygenase type 2 (COX-2), Interleukin-10 (IL-10), and Tumor necrosis factor alpha (TNF) in patients with Crohn’s Disease (CD) and Ulcerative Colitis (UC) between normal mucosa (white circles) and inflamed mucosa (grey circles). Significant differences in gene expression between NAM (Nor-mal Appearing Mucosa) and IM (Inflamed Mucosa) are expressed with “*” equal to a p-value of at least less than 0.05. Data are shown as medians and interquartile ranges. non-significant (p > 0.05).

In CD, as shown in **Figure 2**, in the IM, expression levels of TNF-α (p < 0.0001) and COX-2 (p < 0.0001) at baseline (i.e., T0) were significantly higher than those observed in NAM. In contrast, expression levels of IL-10 (p = 0.001) were significantly lower in IM than in NAM. The same behavior was shown in UC for TNF-α (p < 0.0001) and COX-2 (p < 0.0001) (see **Figure 2**). The expression of IL-10 was lower in IM than in NAM, but this did not reach statistical significance (p=0.065; 95% CI 0.061 – 0.071).

In our *ex vivo* model, the data at T0 reproduce, as expected, an increased inflammatory burden in colonic mucosal tracts with SES-CD or ESS compatible with disease activity (i.e., IM).

**Figure 3:**
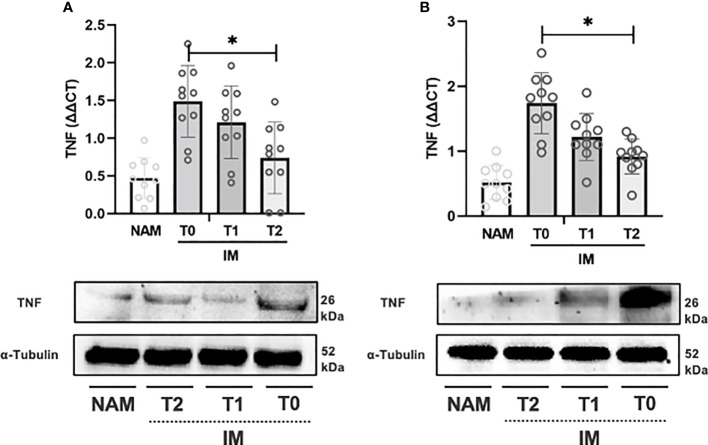
RT-PCR gene expression (above) expressed as expression ratio as well as representative Western Blot (below with control of α-Tubulin) for Tumor necrosis factor alpha (TNF) in patients with Crohn’s Disease **(A)** and Ulcerative Colitis **(B)**. Data are shown for both Normal Appearing Mucosa (NAM) as well as Inflamed Mucosa (IM) both at baseline (T0) and after 120 minutes (T1) and 180 minutes (T2) of incubation with the compound under study. Significant differences in gene expression between times are expressed with “*” equal to a p-value of at least less than 0.05. Data are shown as medians and interquartile ranges.

We then assessed whether HBQ-Complex*^®^* (with the addition of niacin and biotin) was able to affect tissue levels of inflammatory mediators in the IM at different time points. To this purpose, IM in an *ex vivo* preparation was incubated with the compound for up to 180 minutes. COX-2, TNF-α, and IL-10 mRNA levels were determined at T0, 120 minutes (i.e., T1), and 180 minutes (i.e., T2). IM incubation with the compound caused a significant change in the expression of COX-2, TNF-α, and IL-10 at T1 and T2 compared with T0. In CD samples (see **Figure 3A**), TNF-α expression significantly decreased from T0 to T1 and T1 to T2 (p=0.002; 95% CI 0.001 – 0.003).

**Figure 4:**
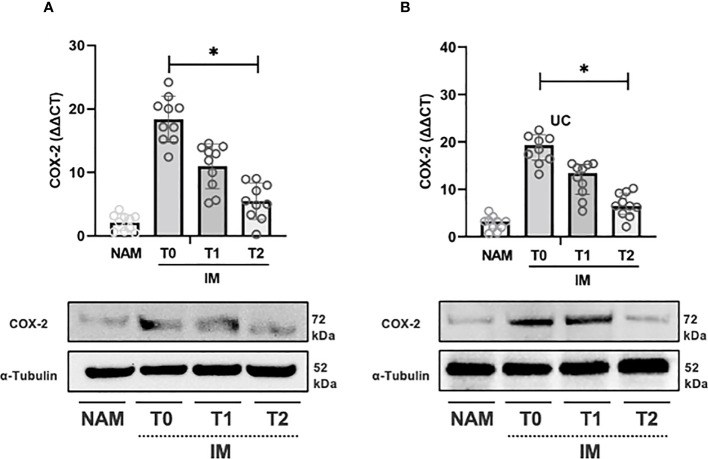
RT-PCR gene expression (above) expressed as expression ratio as well as representative Western Blot (below with control of α-Tubulin) for Cyclooxygenase type 2 (COX-2) in patients with Crohn’s Disease **(A)** and Ulcerative Colitis **(B)**. Data are shown for both Normal Appearing Mucosa (NAM) as well as Inflamed Mucosa (IM) both at baseline (T0) and after 120 minutes (T1) and 180 minutes (T2) of incubation with the compound under study. Significant differences in gene expression between times are expressed with “*” equal to a p-value of at least less than 0.05. Data are shown as medians and interquartile ranges.

**Figure 5:**
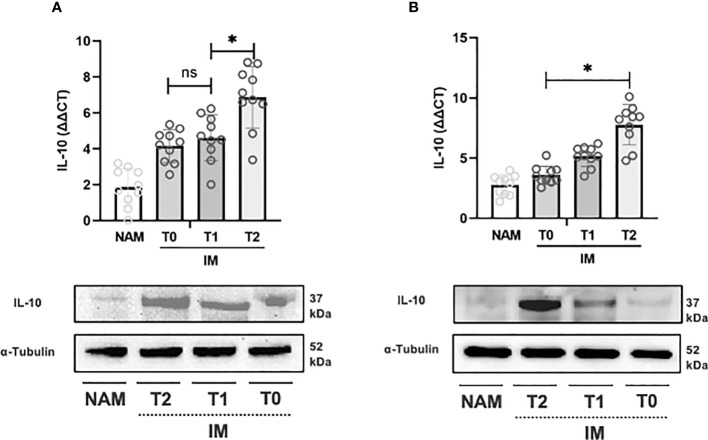
RT-PCR gene expression (above) expressed as expression ratio as well as representative Western Blot (below with control of α-Tubulin) for Interleukin-10 (IL-10) in patients with Crohn’s Disease **(A)** and Ulcerative Colitis **(B)**. Data are shown for both Normal Appearing Mucosa (NAM) as well as Inflamed Mucosa (IM) both at baseline (T0) and after 120 minutes (T1) and 180 minutes (T2) of incubation with the compound under study. Significant differences in gene expression between times are expressed with “*” equal to a p-value of at least less than 0.05. Data are shown as medians and interquartile ranges. non-significant (p > 0.05).

The same pattern was observed for COX-2 expression from T0 to T1 and T1 to T2 (p=0.002; 95% CI 0.001 – 0.003) (see **Figure 4A**). IL-10 expression, on the contrary, increased from T0 to T1 (p=0.064; 95% CI 0.059 – 0.069), but the increase from T1 to T2 was more pronounced (p=0.002; 95% CI 0.001 – 0.003) (see **Figure 5A**).

In UC samples, TNF-α expression decreased from T0 to T1 (p=0.002; 95% CI 0.001 – 0.003) and from T1 to T2 (p=0.004; 95% CI 0.003 – 0.006) (see **Figure 3B**). Likewise, COX-2 gene expression decreased from T0 to T1 (p=0.002; 95% CI 0.001 – 0.003) and from T1 to T2 (p=0.002; 95% CI 0.001 – 0.002) (see **Figure 3B**). IL-10 expression, on the other hand, increased from T0 to T1 and from T1 to T2 (p=0.003; 95% CI 0.002 – 0.004) (see **Figure 5B**).

### 3.3 HBQ-Complex® (in combination with biotin and niacin) downregulates the protein expression of TNF-α and COX-2 and upregulates the protein expression of IL-10

Incubation of colonic samples obtained from the IM of CD and UC patients with the HBQ-Complex*^®^* (in combination with the addition of niacin and biotin) resulted in a progressive decrease in TNF-α and COX-2 protein expression at both T1 and T2 compared with T0 (see **Figures 3**–**5**). As expected, the lowest COX-2 and TNF-α expression was found in the NAM. Conversely, IL-10 tissue expression increased with a maximum peak presence at T2 (see **Figures 3**–**5**).

## 4 Discussion

This is, to date, the first study investigating the *ex vivo* anti-inflammatory potential in IBD patients’ intestinal mucosa of HBQ-Complex*^®^* (with the addition of niacin and biotin), considering the previous experience with *H. erinaceus*, berberine quercetin, and niacin being mainly based on mouse colitis models (7, 8, 15, 16).

In both CD and UC intestinal mucosa samples, this compound induced downregulation of proinflammatory genes (i.e., TNF-α and COX-2) and up-regulated anti-inflammatory genes (i.e., IL-10). Western blot analysis showed agreement results.

Modulation of TNF-α is relevant because the NF-κB family of nuclear transcription factors plays a crucial role in the dysregulation of cytokine production involved in IBD pathogenesis (24, 25). Activation of NF-κB is, moreover, particularly marked in epithelial isolates from bowel specimens obtained from IBD patients with active disease, and its increased expression correlates with the degree of inflammation (26). Moreover, activating this transcription factor increases TNF-α production (26). Not surprisingly, in our study, TNF-α expression was markedly higher in IM than in NAM.

Diling et al. (6), in a mouse model, as previously written, examined polysaccharide extract of *H. erinaceus* and showed remarkably similar results to ours (i.e., in terms of up-regulation of IL-10 and downregulation of NF-κB p65/TNF-α) obtained in a human *ex vivo* study. Ren et al. also recorded similar results under the same conditions with the downregulation of NF-κB (7).

However, the precise mechanism by which *H. erinaceus* up-regulates anti-inflammatory cytokines and down-regulates proinflammatory cytokines is still unclear. This might be contributed to by its antioxidant activity in the gut (7). In detail, Wang et al. (9) weighed the ability of a polysaccharide extract (i.e., EP-1) obtained by precipitation in ethanol after hot water treatment of *H. erinaceus* mycelium to counteract oxidative stress in both an *in vivo* model of murine colitis (induced by acetic acid) and in Caco-2 cells cultures. They recorded a reduction in superoxide dismutase (SOD) activity with an opposite increase in malondialdehyde [a well-known lipid peroxidation marker (27)] after EP-1 treatment of the rats. These results showed EP-1’s ability to exert a scavenging effect toward oxygen free radicals produced in rat’s bowel with acetic acid-induced IBD. EP-1 treatment also improved the morphological changes that acetic acid generated in the rats’ mitochondria, with a recovery of polarization of mitochondrial membrane potential (previously reduced by 70% after acetic acid treatment). The authors, in Caco-2 cells, also confirmed these benefits on oxidative stress (induced by H_2_O_2_) with a decrease in SOD activity, an increase in malondialdehyde, and an improvement in oxygen consumption rate, expressing an improvement in mitochondrial respiration. A further pre-clinical study employing ethanolic extracts of *H. erinaceus* in murine experimental colitis induced by oral dextran sulphate sodium also recorded an upregulation of nitric oxide, malondialdehyde, and superoxide dismutase (28). In addition, within the HBQ-Complex^®^ constituents, antioxidant potential in intestine inflammation has also been observed for quercetin (29, 30) and berberine (31).

An additional mechanism called into play is the modulation of the gut microbiota. *H. erinaceus* can promote a shift in the microbiota toward an increased abundance of short-chain fatty acid (SCFA)-producing bacteria and a relative reduction in pathobionts. Because of these properties, it has often been referred to as a prebiotic or probiotic (6, 10, 32, 33).

In detail, it appears that *H. erinaceus* can promote a selection in the gut microbiota of SCFA-producing bacteria (34). This was observed in healthy volunteers subjected to a dry powder administration of this fungus with an increase in the gut microbiota of the relative abundance of *Bifidobacterium* and *Bacteroides* as well as SCFA-producing bacteria such as *Roseburia faecis*, *Faecalibacterium prausnitzii*, or *Eubacterium rectale* also reducing pathogenic strains (e.g., *Streptococcus thermophilus* or *Roseburia intestinalis*) (10). Furthermore, these microbiota-modifying capabilities were also assayed and demonstrated in mice where *H. erinaceus* increased the relative abundance of butyrate-producing bacteria (i.e., *Lachnospiraceae* and *Ruminococcaceae*) (35).

Moreover, quercetin, berberine, niacin, and biotin, individually, also appear to have anti-inflammatory actions in IBD (15, 16, 18, 19). Quercetin, for example, has been shown to benefit in experimental murine ileitis via a reduction in NF-κB-mediated TNF-α gene expression (36). Furthermore, Bian et al. (37) reported similar results (i.e., downregulation of NF-κB) for berberine in a cellular model of intestinal microcirculation endothelial cells undergoing inflammation by the bacterial lipopolysaccharide. In addition, the downregulation of COX-2 and TNF-α was also demonstrated in a mouse model of experimental colitis after treatment employing other flavonoids as those found in apple polyphenol extract (38, 39). Therefore, we speculate that the anti-inflammatory effects observed in our study may also result from a possible synergistic action among the different constituents of the compound under study.

The anti-inflammatory effect of the this compound was time-dependent, with a progressive decrease of COX-2 and TNF-α and an increase in IL-10 over time from T0 to T2 in the IM. In Western Blot, at T2 in IM, the protein expression of COX-2, TNF-α, and IL-10 were, if not wholly superimposable, nevertheless comparable to that of NAM. We hypothesize, however, that direct exposure to the compound in incubation did not result in perfect attainment of the typical proinflammatory mediators’ expression levels of NAM in the IM mucosa at T2 because this compound, being a nutraceutical, may not possess the target capacity that can probably be offered by other specifically pharmacological agents such as monoclonal antibodies.

Our study is further corroborated by the fact that *H. erinaceus* in *Cynomolgus* monkeys with UC can improve the nutritional status and attenuate UC, decreasing the inflammatory burden and positively affecting gut microbiota (40). It appears that the strains most associated (in pre-clinical studies) with this action and positively modulated are *Lactobacillus reuteri*, *Bacteroides*, *Bifidobacterium*, *Prevotella*, *Parabacteroides*, *Coprococcus*, and *Desulfovibrio*) (32, 40). Conversely, those negatively modulated are *Streptococcus lutetiensis*, *Corynebacterium*, *Staphylococcus*, *Ruminococcus*, *Roseburia*, *Dorea*, and *Sutterella* (32, 40).

When comparing the studies evaluating the beneficial effects of quercetin, berberine, niacin, and biotin with our research, one must keep in mind that our experimental model consists of an *ex vivo* model with tissue obtained from IBD patients. In contrast, most studies with the above nutraceuticals have been conducted in murine experimental models of colitis. Also, we used a combination of all the above nutraceuticals. Finally, in the *in vivo* studies, the different nutraceuticals were administered for several days, whereas in our study, the incubation time of the compound under study was up to 180 minutes.

HBQ-Complex*^®^* has never been tested in IBD patients to assess its impact on clinical, biochemical, endoscopic, and histological features. However, it has been evaluated in symptomatic uncomplicated diverticular disease (SUDD) as a nutraceutical compound showing promising results in inducing clinical remission and fecal calprotectin reduction (41). Considering our observations, we may speculate that the clinical improvement observed in SUDD might be contributed to at least in part by its anti-inflammatory properties. Interestingly, in SUDD, it has been shown that there is an increased expression of TNF-α compared with healthy subjects (42).

Our data should also be evaluated, taking into consideration that our patients were naïve to any IBD treatment, which, conversely, could have resulted in a bias because any therapy the patient had for IBD (such as mesalazine) could have contributed to the change in mediators that were the subject of our study.

This study has several limitations. First, one of the limitations of this study is that we did not explore the mechanisms underlying the anti-inflammatory effect of HBQ-Complex*^®^*. Therefore, further studies are needed to dissect at the molecular level the mechanism whereby this nutraceutical can downregulate inflammation in this *ex vivo* model of IBD. Our pilot work is affected by the limitation of having a small sample size which also necessitates desirable external validation by studies with larger sample sizes to increase the statistical power of our model. In addition, it might be useful to follow the eventual change in cytokines studied in this work in an *ex vivo* setting of IM biopsies incubated without the compound under study to compare with the impact of it. In this regard, we only have data from four samples obtained from four patients of IM mucosa incubated for up to 120 minutes in the absence of HBQ-Complex^®^, niacin, and biotin and in medium only, and no significant changes in the cytokine levels we evaluated (i.e., TNF-α, COX-2, and IL-10) were recorded (*data not shown*). Moreover, another limitation of our model, even if we assessed the variables in a short time frame (i.e., hours), is related to the absence of a metabolism monitoring of the incubated cells (desirable if future studies will assess the compound under study in *ex vivo* longer time evaluation). Finally, other time frames (i.e., < 120 minutes and > 180 minutes) may be assayed in future studies to weigh if the anti-inflammatory modifications induced by HBQ-Complex*^®^* became clear earlier and lasted more than three hours.

In summary, HBQ-Complex^®^ (a mixture of berberine, quercetin, and *H. erinaceus*) in combination with biotin and niacin, downregulates the proinflammatory cytokine TNF-α and COX-2 and up-regulates the anti-inflammatory cytokine IL-10 in an *ex vivo* experimental model consisting of inflamed tissue obtained from patients with CD or UC. Given the increasing interest in the therapeutic potential of nutraceuticals in IBD (43), we speculate that it could be worthwhile exploiting the possibility that HBQ-Complex*^®^* may exert a beneficial effect alone or in combination with well-established therapeutic agents in patients with IBD. Further studies are needed to confirm these findings and to weigh whether HBQ-Complex*^®^* (with the addition of niacin and biotin) may have translational benefits in the clinic of patients with IBD, weighting it by parameters already validated in IBD management (23).

## Data availability statement

The original contributions presented in the study are included in the article/supplementary material. Further inquiries can be directed to the corresponding author.

## Ethics statement

The studies involving human participants were reviewed and approved by Ethics Committee of the University of Campania “Luigi Vanvitelli” (protocol code 15915, 21 May 2021). The patients/participants provided their written informed consent to participate in this study.

## Author contributions

Study concept and design: AG, RP, MR and AF. Acquisition of data: AG, RP, GP, AC, GB, CT, FC, MR, and AF. Formal analysis: AG, RP, and CT. Interpretation of data: AG, RP, GP, AC, GB, CT, FC, MR, and AF. Drafting of the manuscript: AG, RP, GP, AC, GB, CT, FC, MR, and AF. All authors contributed to the article and approved the submitted version.
